# Supplementary material for: Informal caregivers and assistive technology in Norwegian nursing homes
Source: PLoS One. 2022 Oct 4;17(10):e0275474. doi: 10.1371/journal.pone.0275474 (PMC9531810; doi:10.1371/journal.pone.0275474)
Supplement: Attachment — Submitted filename: Response to Reviewers.docx [file pone.0275474.s001.docx]

Dear Academic Editor

Simone Borsci, Ph.D
PLOS ONE

Thank you for your and your reviewer`s comments which make it possible to improve this manuscript, ID PONE-D-22-07145. Below you will find a point-by-point reply to all queries raised by the reviewers. We have also attached the marked-up version with all track changes visible, and one clean copy without visible track changes.

Word count 6244 without references.

Subject: PLOS ONE - Decision on Manuscript ID PONE-D-22-07145

| **Comments** | **Answers** |
| --- | --- |
| **Comments to the Author** |  |
| 1. Is the manuscript technically sound, and do the data support the conclusions?  The manuscript must describe a technically sound piece of scientific research with data that supports the conclusions. Experiments must have been conducted rigorously, with appropriate controls, replication, and sample sizes. The conclusions must be drawn appropriately based on the data presented. Reviewer #1: Partly Reviewer #2: Yes | #1 Thank you for reminding  us to strengthen the manuscript by carefully answering the editor and the reviewer’s questions and comments. |
| 3. Have the authors made all data underlying the findings in their manuscript fully available?  The [PLOS Data policy](https://eur02.safelinks.protection.outlook.com/?url=http%3A%2F%2Fwww.plosone.org%2Fstatic%2Fpolicies.action%23sharing&data=05%7C01%7Clihov%40oslomet.no%7C7afd2d3c209c445dd24008da340b4c18%7Cfec81f12628645508911f446fcdafa1f%7C0%7C0%7C637879521411985309%7CUnknown%7CTWFpbGZsb3d8eyJWIjoiMC4wLjAwMDAiLCJQIjoiV2luMzIiLCJBTiI6Ik1haWwiLCJXVCI6Mn0%3D%7C3000%7C%7C%7C&sdata=S6fMtjERZzMMKFAM%2FWoZPIO48BDFjvEIMD6TqPx0IPs%3D&reserved=0) requires authors to make all data underlying the findings described in their manuscript fully available without restriction, with rare exception (please refer to the Data Availability Statement in the manuscript PDF file). The data should be provided as part of the manuscript or its supporting information, or deposited to a public repository. For example, in addition to summary statistics, the data points behind means, medians and variance measures should be available. If there are restrictions on publicly sharing data—e.g. participant privacy or use of data from a third party—those must be specified. Reviewer #1: No Reviewer #2: Yes | #2 We fully agree that the data should be provided as part of the manuscript or its supporting information. However, this is few interviews in a small municipality in Norway. There are statements from the participants that can be identified. Due to ethical reasons, we do not add the datafile to this manuscript.  See #19, #20 |
| Is the manuscript presented in an intelligible fashion and written in standard English?  PLOS ONE does not copyedit accepted manuscripts, so the language in submitted articles must be clear, correct, and unambiguous. Any typographical or grammatical errors should be corrected at revision, so please note any specific errors here. Reviewer #1: Yes Reviewer #2: Yes | #3 Thank you. |
| **Reviewer 1** |  |
| First of all I read the data availability statement regarding the authors not providing the full transcript of interviews and this seems reasonable from an ethical point of view, as since this was agreed with the participants. The many extracts relevant to the findings are included in the paper. However I would like the interview schedule to be included as supplementary material. | #4 Thank you for asking about the interview schedule. We have added this as supplementary material. See #2. |
| The definition of AT on p.2 is broad and this is OK, but I think the paper could do more to provide a taxonomy, or at least a narrative summary, of the different ATs being talked about. Some of these are more obviously AT than others. Is a chip under the skin, smart lighting or remote controls considered an AT to most readers? They can be of course, by their application, but I think it would be helpful to say somewhere that some of the technologies discussed is conventional AT, some is smart home tech etc. A paper reviewing the types of AT used in dementia care could usefully be included in the citations and refer to the types of AT in the introduction. | #4 Thank you for valuable comments.  Technologies to support older adults and citizens with disabilities can be divided into four domains: 1) for safety and security; 2) for coping with independent living; 3) health technologies; and 4) for well-being (1).  Several terms are used in the literature and in healthcare policy to frame technology for supporting people in need of care, such as, assistive technology (2, 3), assisted living technology (4), telecare (5), eHealth (6), intelligent assistive technology (7), innovative assistive technology (8), ambient assistive living (9) and everyday technology (10, 11).  The translation of terms is challenging, but we use the internationally accepted term *assistive technology* in this study, which is in line with WHO’s policy.  The term everyday technology was introduced in Sweden to cover household aids, electric tools, computers, TVs, mobile phones, ticket vending machines and self-service check-in at airports and hotels (10).  In research, as in clinical practice, it is wise to distinguish between different types of assistive technology and their potential. Gibson (2016) suggested a distinction between technology used *by*, *with* and *on* people with dementia (12):  Such distinctions may be important for both researchers and for healthcare services when identifying suitable technology for assessed needs. However, it is not reasonable to expect that the informal caregiver kept this distinguish in mind when we asked about technology.  We therefore clarify in the term technology in the introduction and in the discussion using Gibson (2016).  We have added more information about the technology on p 2, 13, 14 |
| The paper does not explain one very important thing. It is not explained in the paper if and how the interviewees were prompted about technologies. In the discussion the statement 'None of the informal caregivers mentioned the use of more recent ATs' suggested they were prompted as the extracts do show that the more recent ATs were mentioned. The interview schedule should be included and all of the prompts by the research team given. Then, it should be made a lot clearer which extracts are spontaneous mentions of technologies and which were prompted by the researchers. If not it is hard to assess the level of knowledge of the interviewees and I have a concern that the caregivers were led too much by the researchers. In hindsight it might have been a good idea to have asked about different types of AT in a systematic way, to find out the level of knowledge about them. | #5 These comments allow us to improve the manuscript. We have carefully considered others quotations about technology. We have added comments from interview no four. However, not from Informal caregiver no 11. He was together with his wife in the interview, and did not add anything of interest for this study. All the interviews are presented in then findings. See also #2 and #20.  However, we asked broadly about technology and not asking about what was available at the nursing home. It was of importance to explore the informal caregivers` ideas about technology based on their knowledge and experiences. This approach may result in different types of technology being mentioned. See #4  We have added the interview schedule, see #2 and #4 |
| The Conclusion should likewise be clearer about 'little or no knowledge' as it appears from the interviews that even if caregivers were not familiar with using more recent ATs they do have opinions about them. In the limitations, the statement that "perspectives were based solely on assumptions about AT" seems to me to be rather judgemental. From the extracts of the interviews, it appears that some caregivers did have direct experience of some technologies having been used in the nursing homes by their relatives (such as the example of the exercise bike, and the watch that was lost). So I am not sure it is fair to call this a limitation of the study. I think this assessment of knowledge would be better placed in the discussion and for the assessment not be so black and white about this point. | #6 Thank you, we have changed the conclusion.  See p 17 |
| In the results, page 8, when the quote is given about caregiver 1 'she felt that she was seen as a problem', can it be clearer who they are problem to? I assume is it the nursing home staff but this is not specified. | #7  This is rewritten. |
| The section headings and subheadings are explanatory but I would prefer to have some hierarchy so that METHODS and RESULTS are top level headings, with subheadings underneath. DISCUSSION is given in capitals as are CONCLUSIONS so this style could be followed earlier. | #8  All amended |
| Typographical errors/suggestions for rewording:  Abstract: Background - 'collaborators for the staff' -> 'collaborators with staff'; Results - 'Informal caregivers are positive to' -> 'Informal caregivers were positive about' | #9 All admitted |
| Introduction: para. 1: 'have a dementia disease' -> 'have dementia' or 'have a dementia diagnosis'; para 2: 'for the healthcare service' -> 'for healthcare services'; para. 3 'express this' -> 'express these'. | #10  1: 'have a dementia disease' is rewritten: 'have dementia'  2: rewritten  3: rewritten |
| Data analysis: reference (22) is Graneheim, Lindgren, Lundman (or use et al.) | #11  Rewritten: Graneheim et al. |
| Ethical considerations: NSD acronym needs to be expanded unless this is already in the redacted content. | #12 This is already in the redacted content. |
| Discussion: the point about sample sizes should be moved to limitations? | #13 Done, see p 17 |
| The wording 'coercive clause' is unusual - can this be reworded. I think you mean 'coercive practice was used' and/or use the word 'containment'? | #14  'coercive clause' is rewritten ‘coercive practice was used', p 15 |
| The sentence with 'no automaticity in ATs' is hard to follow and this could be reworded. I think you mean that using ATs does not remove the need for a person to be involved in the process of care | #15 The sentence is omitted |
| **Reviewer 2** |  |
| 1. Authors should clarify why only one ward was included from the 2nd nursing home. Was this the only ward for people with dementia? | #16 Done, see p 4. |
| 2. To give the readers a better sense of what was asked during the interviews, authors should provide the interview schedule and follow-up questions in supplemental material. | #17, See #2 and #4 |
| 3. Table 2: not all readers will be familiar with the stated strategies. Authors should provide more detailed information about these strategies and their implementation in supplemental material. | #18, Amended, please see additional information in table 2. |
| 4. In the Preunderstandings section, authors state that preunderstandings and prejudices were debated ahead of the analysis process. It is not clear what the result of this debate/discussion was and how that minimized bias in analyzing the interviews. | #19 – Amended, please see p. 6. |
| 5. Authors include statements by 9 of the 11 informal caregivers. One is quoted 3 times. Authors may want to see if they can include quotes from each of the caregivers. | #20 We have added information from all the interviews, p 7 and . See #2, and #5. |

1.	NOU 2011:11. Innovasjon i omsorg (Innovation in the Care Services). Ministry of Health and Care Services (Helse- og omsorgsdepartementet).

2.	WHO. Assistive technology. 2018 [Available from: <https://www.who.int/news-room/fact-sheets/detail/assistive-technology>.

3.	WFOT. Position Statement: Occupational Therapy and Assistive Technology 2019 [Available from: <https://www.wfot.org/resources/occupational-therapy-and-assistive-technology>.

4.	Forsberg E-M, Thorstensen E, Casagrande FD, Holthe T, Halvorsrud LT, Lund A, et al. Is RRI a new R&I logic? A reflection from an integrated RRI project. 2020.

5.	Berge MS. Telecare acceptance as sticky entrapment: A realist review. Gerontechnology. 2016;15(2):98-108.

6.	Jakobsson E, Nygård L, Kottorp A, Malinowsky M. Experiences from using eHealth in contact with health care among older adults with cognitive impairment. Scand J Caring Sci. 2019;33:380-9.

7.	Ienca M, Wangmo T, Jotterand F, Kressig RW, Elger B. Ethical Design of Intelligent Assistive Technologies for Dementia: A Descriptive Review. Science and engineering ethics. 2018;24(4):1035-55.

8.	Thordardottir B, Malmgren Fänge A, Lethin C, Rodriguez Gatta D, Chiatti C. Acceptance and Use of Innovative Assistive Technologies among People with Cognitive Impairment and Their Caregivers: A Systematic Review. BioMed Research International. 2019;2019.

9.	Nordic Innovation and Nordic Welfare Centre. Nordic ambient assistive living. Welfare technologies for active and independent living at home. 2019.

10.	Nygård L, Rosenberg L, Kottorp A, inventorsEveryday Technology Use Questionnaire - ETUQ. Sweden2015 25.01.2016.

11.	Malinowsky C, Kottorp A, Nygard L. Everyday technologies' levels of difficulty when used by older adults with and without cognitive impairment-Comparison of self-perceived versus observed difficulty estimates. Technology and Disability. 2013;25(3):167-76.

12.	Gibson G, Newton L, Pritchard G, Finch T, Brittain K, Robinson L. The provision of assistive technology products and services for people with dementia in the United Kingdom. Dementia (London). 2016;15(4):681-701.
