# Supplementary material for: Informal caregivers and assistive technology in Norwegian nursing homes
Source: PLoS One. 2022 Oct 4;17(10):e0275474. doi: 10.1371/journal.pone.0275474 (PMC9531810; doi:10.1371/journal.pone.0275474)
Supplement: Attachment — Submitted filename: Response to reviewers.docx [file pone.0275474.s002.docx]

| Comments | Answers |
| --- | --- |
| 'them self' in the addition about Gibson in the DISCUSSION should be 'themself'. Also 'That used on persons with dementia were devices and systems used by informal or formal caregiver to care for a person with dementia; examples are monitoring systems, environmental sensors, cameras, alarms and so on.' I would suggest instead: Technologies used on persons with dementia were devices and systems used by informal or formal caregivers to care for a person with dementia; dementia; examples are monitoring systems, environmental sensors, cameras and alarms.' (add 'Technologies' and 's' to caregiver, remove 'and so on' as these are given as examples so others would be expected). | Amended in accordance with the suggestions |
| Also in DISCUSSION: 'it was decided to use a coercive practice was used and locking residents inside their rooms.'  needs correcting to 'it was decided to use a coercive practice whereby residents were locked inside their rooms.'  or (I would prefer): 'it was decided to contain residents by locking them inside their rooms. | Changed to 'it was decided to contain residents by locking them inside their rooms. |
| In LIMITATIONS: 'First, the small sample size (11 participants) limits the findings to similar populations and contexts.' I would put (11 participants in 2 care homes) Also in LIMITATIONS I think 'did not have any direct experience of it' would be better. | Amended according to the suggestions |
| Reviewer #2: I did not see the supplemental material which supposedly includes the interview schedule and follow-up questions. Authors should be sure that it is included and refer the reader to the supplement. | The interview guide was uploaded during the submission process. The reader is refered to the supplement at suggested with the following sentence: see supplement for interview schedule |
